# Feedforward control in children with cerebral palsy and association with white matter integrity

**DOI:** 10.3389/fneur.2025.1515432

**Published:** 2025-03-07

**Authors:** Ophélie Martinie, Philippe Karan, Martin Simoneau, Maxime Descoteaux, Catherine Mercier, Maxime T. Robert

**Affiliations:** ^1^Center for Interdisciplinary Research in Rehabilitation and Social Integration (Cirris), CIUSSS de la Capitale-Nationale, Quebec City, QC, Canada; ^2^School of Rehabilitation Sciences, Laval University, Quebec City, QC, Canada; ^3^Department of Computer Sciences, Université de Sherbrooke, Sherbrooke, QC, Canada; ^4^Department of Kinesiology, Laval University, Quebec City, QC, Canada

**Keywords:** tractography, diffusion neuroimaging, precision grip, anticipatory control, motor planning

## Abstract

**Background:**

Precise upper limb movements required for daily activities rely on feedback and feedforward control mechanisms. In children with cerebral palsy (CP), damage to white matter tracts impairs motor execution and sensorimotor control. Most studies in CP have focused on motor execution deficits, whereas the relationship between feedforward control alterations and white matter microstructure features has received less attention.

**Method:**

This study compared feedforward control during a grasp and lift task in 9 children with CP (diagnosis of hemiplegic CP with mild to moderate upper limb deficits) to 40 typically developing (TD) children aged 8 to 17 years. A secondary objective was to examine associations between feedforward control and the microstructural measures of corticocerebellar and other motor-planning related tracts. All participants completed 13 trials of the grasp and lift task. The CP group also underwent diffusion magnetic resonance imaging (MRI) using a 3-Tesla system to acquire anatomical and diffusion MRI.

**Results:**

Results showed feedforward control deficits in the non-dominant hand of children with CP, reflected by reduced peak force rates before sensory feedback was available and a lack of adaptation across trials. Strong correlations were observed between feedforward control and microstructural measures of the corticospinal tract and superior longitudinal fasciculus, but not with the corticocerebellar tracts.

**Conclusion:**

These findings suggest that broader sensorimotor processes, beyond feedforward control alone, contribute to force control deficits observed in children with CP.

## 1 Introduction

Most daily activities require coordinated use of the upper limb and digits (1). Even trivial tasks such as grasping a glass involve prior knowledge of object’s properties and integration of sensory information from the environment (e.g., texture, gravity) for optimal manipulation (2). This coordination relies on the feedback and the feedforward control [for a recent review on this topic, see (3)], which, respectively, allow online movement correction based on sensory information and supports the planning of force control, enabling the selection of movement parameters based on the task goal (4, 5). Feedforward control builds motor representation (6) by refining internal models that associate movement with sensory outcomes, adapting forces along trials in grasp and lift tasks (7, 8). Deficits in feedforward control may lead to sensorimotor deficits or exacerbate them in neurological disorders, for instance, children with cerebral palsy (CP) (2, 9).

CP is characterized by a group of sensorimotor symptoms affecting posture and movement due to alterations of the developing brain in the fetus or up to one to two years after birth (10). The lesions affect a vast distributed network, leading to impairments in motor control at both planning and execution levels (9, 11, 12). Children with CP show feedforward control deficits compared to their typically developing (TD) aged-matched peers, particularly in force control [for a review, see Martinie et al. (13)]. In grasp and lift tasks, children with CP show a sequential increase in load and grip forces [LF and GF; Eliasson et al. (14)] and fail to adapt to object weight and texture (15), resulting in a multi-peak force profile instead of a smooth, coordinated grasp force (16). They also exhibit inter-trial variability in their force profiles, while TD children stabilize their force after two trials (17). The feedforward force control deficits are only present in the more affected hand in hemiplegic CP suggesting asymmetrical abilities (18). Those asymmetrical deficits are specific to this sensorimotor task in which the less affected hand of children with unilateral CP behaves like the dominant hand of TD children (13, 19, 20).

Diffusion magnetic resonance imaging (dMRI) allows the quantification of water molecules’ movement along physical barriers (e.g., myelin sheath) to reconstruct white matter pathways (21, 22). DMRI studies in children with CP have emphasized the role of corticomotor tracts in their feedforward control deficits (23, 24). However, studies in healthy participants suggest that beyond corticomotor tracts (25), other brain regions are involved in feedforward force control, including the parietal cortex (26) and cerebellum (27–29). These findings point to a widely distributed network that connects motor areas, parietal regions, and the cerebellum, which are engaged in different aspects of force planning. Parieto-frontal pathways play a role in sensorimotor control, involving the internal representation of the body and grasping parameters (30–33). Additionally, these pathways monitor discrepancies between predicted and actual sensory feedback without correcting motor commands directly (34). Such corrections rely on feedforward control mechanisms facilitated by cerebellar afferent connections [e.g., fronto-ponto-cerebellar tracts; Ramnani (35) and Ugolini and Kuypers (36)] and efferent pathways [e.g., cerebello-thalamo-frontal tracts; Ramnani (35) and Ito (37)]. Although corticocerebellar tracts alterations have been linked to poorer manual dexterity in children with CP (38, 39), there is limited understanding of their involvement in feedforward control in CP.

The first objective of this study is to compare feedforward control between children with CP and TD children in a grasp and lift task. The second objective is to assess whether feedforward control is associated with cortico-cerebellar tracts and other motor-planning-related tracts in children with CP. We anticipate that children with CP will exhibit impaired feedforward control with their non-dominant hand (i.e., more affected hand) compared to TD children, but not with their dominant hand. We expect to observe differences in force variables and reduced adaptation (i.e., changes throughout the task). We predict that these differences will be increased for children aged 8 to 11 years, as TD children already have mature adult-like forces peak rate at eight years of age whereas 6 to 8 years old children with CP usually behave like two years old TD children (14) and mature until early adulthood (40, 41). Additionally, we expect to observe less adaptation between pairs of trials as an indication of this lack of adaptation. We also hypothesize that asymmetry in feedforward control between hands will correlate with the asymmetry in the microstructural features of cortico-cerebellar tracts (i.e., fronto-ponto-cerebellar, FPC; cerebello-thalamo cortical, CTF) and with other motor-planning-related tracts, such as the corticospinal tracts (CST) and motor-parietal tracts (e.g., the superior longitudinal fasciculus, SLF), which are involved in the control of grasping and lifting.

## 2 Method

### 2.1 Participants

TD children and children with CP were recruited. Inclusion criteria included: (1) aged between 8 and 17 years old and (2) able to understand verbal instructions. In addition, children with CP were included if: (1) diagnosed with unilateral spastic CP; (2) had a Manual Ability Classification System [MACS, Eliasson et al. (40, 41)] of level I-III (i.e., mild to moderate deficits). Exclusion criteria were: (1) surgery in the upper limb in the past six months, (2) MRI non-compatibility for children with CP (criteria applied for participation in the MRI session only, i.e., the inability to perform the MRI scan did not exclude the participant from the grasp and lift task). The presence of tactile deficits was measured based on the last clinical assessment of the two-point discrimination task (42) available in their medical record. All children and their caregivers signed permission and consent. The study was approved by the CIUSSS-CN Committee (RIS_2024–2031).

### 2.2 Procedure and apparatus

Our experiment was divided into two sessions: (1) behavioral assessment and (2) MRI scan. Both TD and CP children participated in the behavioral session, which consisted of a grasp and lift task. The task consisted of grasping and lifting a 700 g object with sandpaper on the handling loadcell and two force loadcells (6 degrees of freedom, model Nano-17, ATI Instrumentation, Apex, NC, USA) measuring forces in x, y and z axis. The participants were asked to start the task with their non-dominant hand determined with a short child-friendly adapted version of the Edinburgh Handedness Inventory (43). Comfortably seated at arm’s distance from the object, children had to perform a maximum voluntary contraction by grasping the object three times without lifting it, for 5 s. The average of the three trials allowed us to compute the maximum grip force for each hand (Fmax), which was used in a ratio using Fmax non-dominant hand/Fmax dominant hand. Participants were then asked to grasp and lift the object at a comfortable speed. Following light-emitting diode (LED) activation, the participants grasped and lifted the object 13 times. After keeping the object 20 centimeters above the tabletop (i.e., in front of the LED line fixed into a wooden panel behind the object), they moved the object back to its initial position when the LED turned off (i.e., after 6 s).

Only children with CP performed the MRI session in which both structural and diffusion images were acquired. The participants underwent the scan without any sedation. Language-friendly explanation for children was provided about the procedure. To reduce stress, they also could watch a cartoon during the scan. The MRI data were acquired with a 3 T Siemens scanner (Magnetom Prisma, Siemens Healthineers) with a 32-channel head coil. The total time of data acquisition was between 15 to 21 min. The scan session took place at the MRI platform of the CERVO research center (Quebec City, Canada).

### 2.3 Data acquisition and preprocessing

For the grasp and lift experiment, data were acquired and processed using a custom-made MATLAB script (R2021b version, MathWorks, Massachusetts, USA). The GF signal was calculated by summing each loadcell signal along the z-axis, while the LF was calculated by summing each loadcell signal along the y-axis.

For the MRI data, we adhered to the detailed procedure outlined in Martinie et al. (44). However, we have summarized the key steps here. Both structural T1 weighted image (turbo field echo multishot protocol in axial plane, voxel size = 1 mm isotropic, 180 slices, TR = 7.3 ms, TE = 3.3 ms, inversion time = 940 ms, flip angle = 9°) and diffusion images (echo-planar single shot, 60 slices, HARDI sequence detailed in https://zenodo.org/record/2602049, slice thickness = 2 mm, matrix 112 × 112, in-plane resolution = 2 × 2 mm, flip angle = 90°, FOV = 224 × 224 mm, 84 directions, b-values = 300 s/mm2, 1,000 s/mm2 and 2000 s/mm2, 6 b = 0 volumes, TR = 7,650 ms, TE = 89.5 ms) were acquired. The preprocessing and processing steps were performed on Beluga, a computational platform offered by Calcul Quebec (provincial partner of the Digital Research Alliance of Canada, Quebec, Canada, https://alliancecan.ca/en). Each step of preprocessing, signal reconstruction, and tract extraction was meticulously inspected by two authors. In cases of abnormalities or disagreements, a third author with expertise in neuroimaging was consulted for their opinion.

Tractoflow version 2.3.0 pipeline, showing between 98 to 100% reproducibility, [code available at https://github.com/scilus/tractoflow.git, Theaud et al. (45)] was used to correct the data and extract the diffusion metrics (i.e., diffusion tensor imaging, DTI; constrained spherical deconvolution, CSD). In addition, the pipeline contained also a probabilistic whole brain tractography using Particle Filter Tracking parameter based on fiber orientation density function in T1-based white matter map (FA threshold = 0.1, step size = 0.5 mm, maximum angle between 2 steps = 20°, number of seeds per voxel = 10, and stopping criteria = gray matter mask) (44). To evaluate white matter microstructure, dMRI metrics based on DTI and CSD were extracted from the tractoflow output using tractometry_flow (code available at https://github.com/scilus/tractometry_flow.git, Cousineau (46)). In addition, neurite orientation dispersion and density imaging (NODDI) metrics, based on a multi-compartment model (47), were computed using the resampled DWI, the resampled b0, and the bval and the corrected bvec files from preprocessing pipeline, with noddi_flow [code available at https://github.com/scilus/noddi_flow.git, Daducci et al. (48)].

The extraction of cortico-cerebellar tracts was performed using a strategy of tractography based on regions of interest (ROIs) which successfully identified those tracts in CP (44) from the atlas JHU ICBM DWI 1 mm [available at https://neurovault.org/media/images/264/JHU-ICBM-DWI-1mm.nii.gz, Mori et al. (49)], Talairach atlas [available at http://www.talairach.org/, Lancaster et al. (50) and Brainnetome atlas (available at http://www.brainnetome.org/resource/)]. The ROIs were registered to our participants’ space using ANTs (51), and the tractograms were extracted using the Scilpy toolbox (available at https://github.com/scilus/scilpy.git). For the FPC, the included ROIs were premotor cortices, internal capsule, and contralateral middle cerebellar peduncles [for the precise description of the regions of interest, see Palesi et al. (52, 53)]. For the CTF, the included ROIs were superior cerebellar peduncles, ventral and lateral nuclei of the contralateral thalamus and contralateral premotor cortex. For both tracts, the excluded ROIs were corpus callosum and adjacent cortices to avoid errors. Tracts over a length threshold of 120 mm were excluded to avoid spurious streamlines (51).

Other tracts involved in motor planning were extracted using a multi-atlas virtual dissection method called Recobundle X, which allows automatic extraction of white matter bundles based on their shape [available at https://github.com/scilus/rbx_flow.git, Garyfallidis et al. (54) and Rheault (55)], with greater validity and less heterogeneity than classic tract extraction method (56). The chosen tracts were the CST, and an associative frontoparietal tract called the SLF, both known to be involved in motor control (25, 57, 58).

To characterize brain lesions of the CP participants, the lesion volume was computed using the tissue volume ratio in each hemisphere with the following formula: (gray + white matter) left/(gray + white matter) right (59). An important difference in tissue volume between hemispheres was shown with a ratio close to 0. Tissues segmentations were performed using Freesurfer method [code freely available at http://surfer.nmr.mgh.harvard.edu/; Fischl (60)] with a freesurfer_flow (code available at https://github.com/scilus/freesurfer_flow) on registered T1 outputted from Tractoflow.

### 2.4 Variables extraction

The analyses included two measures of feedforward control (i.e., GF peak rate and LF peak rate) and four measures of white matter tracts microstructure (i.e., fractional anisotropy, FA; apparent fiber density, AFD; orientation dispersion, OD) and macrostructure (i.e., volume) for the FPC, CTF, CST, and SLF.

To assess feedforward control, the forces peak rates were used as the peaks of both GF and LF rates occur before sensory feedback about the object’s weight is available Johansson and Westling (17) and Wolpert and Flanagan (61). These variables have been widely employed as measures of feedforward control, especially for children with CP [for review, see Martinie et al. (13) and Bleyenheuft and Gordon (62)]. GF and LF peak rates were computed by taking the maxima of differences between consecutive force values and dividing by the time interval, following the formula F/∆t, where ∆t represents the time interval between two force measurements (17, 63, 64). This procedure was performed using the *diff* function in MATLAB, representing the amplitude and maximum variation of GF and LF forces.

The dMRI metrics used were selected prior to analysis based on their prevalence in the neuroimaging literature on CP (65) and their similarity, despite originating from different models. The. selected DTI-based metric, FA, measures the degree of tissue anisotropy, with the highest values in uniformly oriented tissue, such as white matter. FA is widely used in clinical populations, particularly in CP (65), where it has demonstrated strong reliability (66). FA is sensitive to myelination and membrane permeability (67). However, its sensitivity is limited in the presence of crossing fibers, so complementary metrics are recommended (68). Consequently, from CSD, the total AFD was computed from the fiber orientation distribution function. AFD shares FA’s sensitivity but offers greater specificity, particularly in regions with crossing fibers (67, 69). Given the higher sensitivity of NODDI metrics compared to FA (70) and their increasing use in clinical research (71), the OD was included in this study. The OD reflects the dispersion of fibers within a voxel and is associated with neural reorganization, axonal dispersion, and neuroinflammation (72). Lastly, tract volume was considered as a macrostructural metric (73). These diffusion and volume measures were assigned to the entire tracts by averaging the metrics of all voxels within the white matter pathways, a process automatically performed by the tractometry_flow script used.

The main independent variables in the analysis were three factors with two levels: group (CP vs. TD), hand (non-dominant vs. dominant), and age (younger vs. older). The younger age refers to participants ≤11 years of age, and the older group comprises participants >11 years.

### 2.5 Analysis

Statistical analyses were conducted using RStudio 2023.12.1.402 version (Posit team, 2024). The analyses were divided into feedforward force control in both TD and CP children and the association between feedforward control and white matter microstructural measures in children with CP only. As the assumptions of normality and homoscedasticity were not met, non-parametric tests were employed.

*Objective 1:* Comparison of feedforward control between children with CP and TD children.

The analyses were performed separately for the GF and LF peak rates. Each force variable was tested following four steps: (1) difference of group × age for each hand; (2) tendency of adaptation along trials; (3) amount of change between pairs of consecutive trials; (4) main effect of trials. Due to the limitations of non-parametric tests in assessing interactions among three factors, two-factor analyses were conducted separately for each hand to compare the CP and TD groups while accounting for the potential effect of age. Only the fourth section of the analyses included the factor trial (1 to 13).

(1) Difference between group × age by hand.

To determine whether a global effect of group and age was present for GF and LF peak rates for each hand, the Scheirer-Ray-Hare test, a non-parametric equivalent of the two-way ANOVA, was used. Bonferroni correction was applied to adjust for multiple comparisons. For significant interactions, pairwise post-hoc comparisons were conducted using the Wilcoxon signed-rank test, with Bonferroni correction applied to control for multiple testing.

(2) Tendency of adaptation along trials.

A linear regression slope was computed for each participant to assess whether variables adapt (i.e., change) across trials according to group and age. The slope represents the linear trend and rate of change across trials, indicating the general tendency for adaptation. The slopes were compared between the groups (CP vs. TD) and ages (youngest vs. oldest) using the Scheirer-Ray-Hare test and Bonferonni correction, separately for each hand. If significant interactions were found, Wilcoxon signed-rank post-hoc tests were performed to further investigate specific pairwise differences. Additionally, each slope was tested against zero to determine if a significant change over trials was present.

(3) Amount of change between consecutive pair of trials.

To determine if local trial-to-trial force variations occurred and whether these variations were equally distributed or if the force stabilized (i.e., absence of variation), the relative change between pairs of consecutive trials was computed using the formula: (|trial_2 – trial_1|)/(trial_1) for each pair until the last trial. The relative change of force peak rates was analyzed separately for each hand, incorporating the factors group (CP vs. TD) and age (youngest vs. oldest) using the Scheirer-Ray-Hare test. Significant interactions were further examined using Wilcoxon signed-rank post-hoc tests if necessary. Additionally, the slope of GF peak rate across trials was tested against zero to verify if a significant change over trials existed.

(4) Main effect of trials.

To identify specific differences between trials and assess the main effect of trials, a repeated measures Friedman test was performed for each condition due to the test’s limitation in including all factors simultaneously. Multiple testing corrections were applied. If a main effect of trials was observed, post-hoc pairwise comparisons were conducted using Conover’s test with Holm correction to account for multiple comparisons.

*Objective 2:* Association between feedforward control and white matter tracts.

The association between white matter microstructure features and force control was assessed using Spearman correlations between the FPC, CTF, CST, and SLF. Given the exploratory nature of the analysis and the small sample size, the *p*-value was not corrected, and the results should be interpreted as hypothesis-generating, requiring validation in larger. To account for hand and lesioned side while minimizing multiple testing, asymmetry indices were calculated for hand GF and LF peak rates and hemisphere microstructural measures using the formulas (dominant side – non-dominant side)/(dominant side + non-dominant side) and (contralesional side – ipsilesional side)/(contralesional side + ipsilesional side). The asymmetry indices of each variable were tested against zero using a Wilcoxon signed-rank test.

## 3 Results

### 3.1 Descriptives statistics

Forty TD children (22 girls and 18 boys, *M* = 12.12, SD = 2.63, from 8.1 to 16.9 years old) and nine children with CP (6 girls and 3 boys, *M* = 11.06, SD = 2.01, from 8.38 to 14 years old) were recruited. One participant with CP could not perform the MRI because of the presence of a ventricular shunt. Among the children with CP, 7 had a pre-or peri-natal stroke. Clinical data of children with CP are described in Table 1. TD children had a higher non-dominant/dominant Fmax ratio (M = 1.02, SD = 0.2) than children with CP (*M* = 0.54, SD = 0.21).

**Table 1:** Clinical data for children with CP, including age, sex (F, female; M, male), Fmax hand ratio and tactile deficits for the non-dominant hand.

ID	Age	Sex	MACS	Fmax ratio	Tactile deficits	Lesion side	Etiology	Tissue ratio	AI
43	8.33	F	3	0.43	No	Left	Pre-natal stroke	No segmentation	
8	10.75	F	2	0.30	No	Left	Periventricular leukomalacia	0.77	0.13
36	14	M	1	0.64	No	Right	Stroke (undefined)	1.06	0.03
81	9	F	2	0.48	Yes	Right	Pre-natal stroke	No segmentation	
37	9.9	F	2	1.53	No data	Right	Periventricular leukomalacia	No MRI	
60	13.2	M	1	0.79	No	Right	Pre-natal stroke	1.08	0.04
44	11.6	F	2	0.73	No data	Right	Pre-natal stroke	1.08	0.03
79	9.7	F	2	0.22	No	Left	Post-natal stroke	0.94	0.03
35	13.2	M	1	0.69	No	Left	Pre-natal stroke	1.02	−0.01

The tissue volume ratio and asymmetry index (AI) between the contralesional and ipsilesional hemispheres characterize the extent of the lesion. Segmentation was not possible for two participants because of too many tissue deformations.

#### 3.1.1 Objective 1: comparison of feedforward control between children with CP and TD children

##### 3.1.1.1 GF peak rate

**Figure 1:**
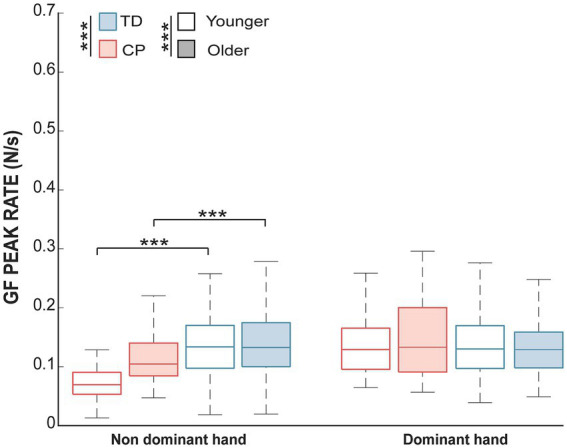
The median and interquartile range of the GF peak rate in each group and each age range. Significant differences for the non-dominant hand for both age groups of the children with CP compared to TD children (adjusted *p*-value <0.001; ***). No effect was found for the dominant hand. A white shade represents children aged between 8 and 11, whereas blue or red shade represents older children.

The **difference** for the GF peak rate was tested for each hand. No significant effects of group or age were observed for the dominant hand. For the non-dominant hand, there were significant effects of both groups (*H* = 75.83, adjusted *p*-value <0.001) and age (*H* = 5.10, adjusted p-value = 0.04), as well as an interaction between these factors (*H* = 9.67, adjusted p-value = 0.004). Pairwise post-hoc comparisons, therefore, examined the effect of groups within each age separately. The GF peak rate was found to significantly differ between groups both in the younger (*W* = 2,407, adjusted p-value <0.001) and older groups (*W* = 5,557, adjusted *p*-value = 0.0006). The Figure 1 illustrates the differences in GF peak rate. A posteriori analyses were conducted to investigate whether the differences in the GF peak rate occurred because of differences in the maximum raw GF during the task or the time to reach the maximum raw GF. Maximum raw GF was not significantly different between CP and TD children (*p*-value = 0.8), but the time to peak was different (*p*-value <0.001), with a longer time to peak for CP (*M* = 2 ms, SD =1.6) compared to TD children (*M* = 1.1 ms, SD = 1.4).

The **adaptation along trials** testing the slope of the GF peak rate for each hand revealed no significant differences between groups or age. Additionally, when the slopes were tested against zero, none of the conditions exhibited a slope significantly different from zero. This observation indicates a general tendency for constancy throughout the task, irrespective of the group, hand, or age.

The **amount of change between pairs of trials** of the dominant hand did not show any significant effects. Significant differences in the GF peak rate relative change were observed for the non-dominant hand, with a main effect of group (*H* = 6.06, adjusted *p*-value = 0.02) and an interaction between group and age (*H* = 9, adjusted *p*-value = 0.003). Post-hoc pairwise comparisons of the effect of the group for each age indicated a significant effect of the group only for the younger participants (*W* = 8,172, adjusted *p*-value <0.001). These results suggest that the relative change is influenced by group and age for the non-dominant hand. Additionally, the slopes of the relative change did not differ from zero for any condition, suggesting no overall trend or significant change in force across trials for any of the conditions.

The **trial factor** did not show any significant effects.

##### 3.1.1.2 LF peak rate

**Figure 2:**
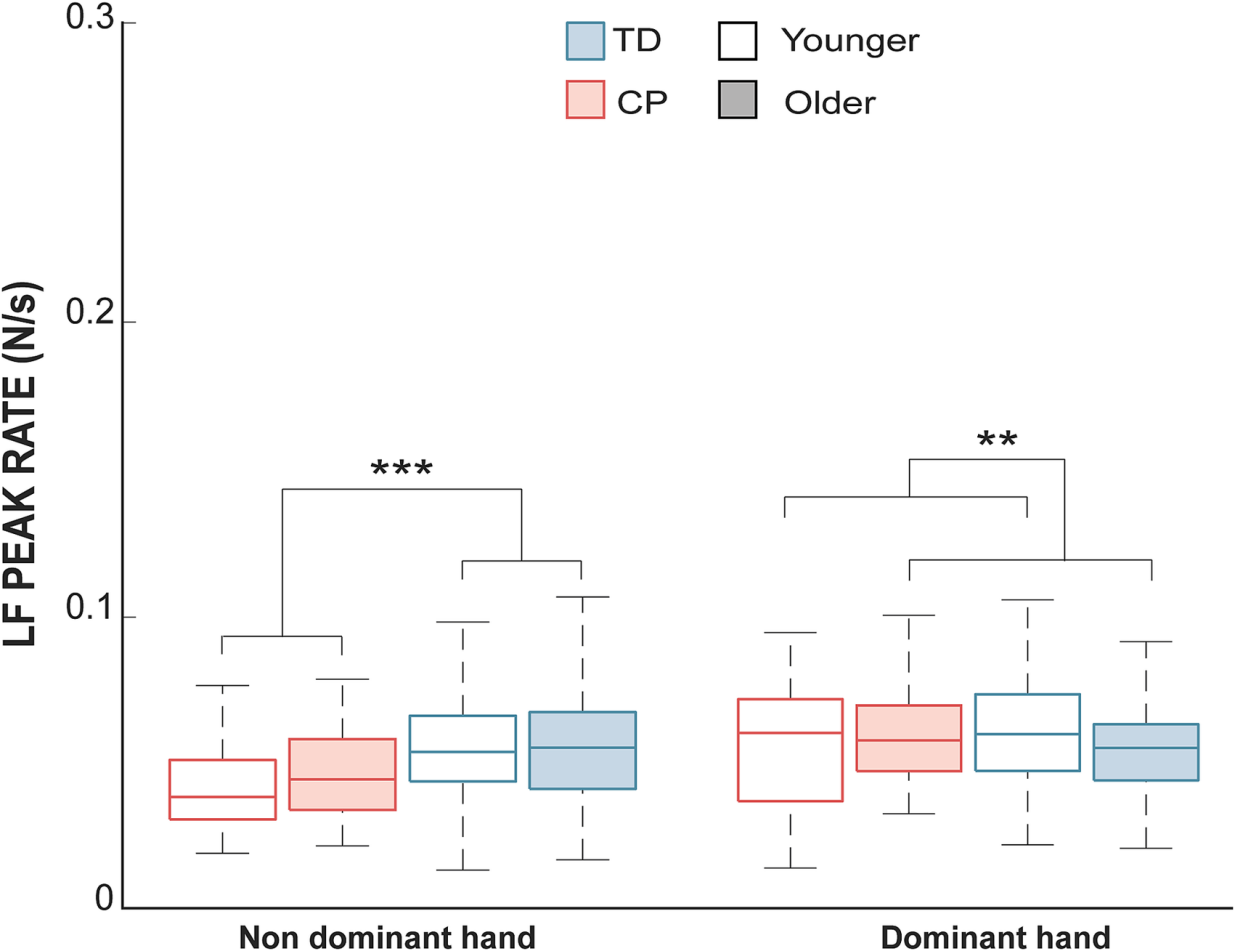
The median and interquartile range of the LF peak rate in each group and each age range. Significant effects of group for the non-dominant hand and age for the dominant hand were found (adjusted *p*-value <0.001, ***; *p* < 0.01, **). A white shade represents children aged between 8 and 11, whereas blue or red shade represents older children.

The **difference** for the LF peak rate was tested for each hand. It revealed a main effect of age (H = 11.09, adjusted *p*-value = 0.004) for the dominant hand, irrespective of the group, as well as a main effect of group for the non-dominant hand (*H* = 41.69, adjusted *p*-value <0.001). This result indicates that the non-dominant hand’s LF peak rate varied between groups. Figure 2 illustrates the group effect for the non-dominant hand and the age effect for the dominant hand. A posteriori analyses were conducted to test whether the differences in the LF peak rate resulted from differences in maximum LF during the task or in time to reach the maximum LF. Both maximum LF and time to LF peak were significantly different between CP and TD children (*p*-value <0.001), with higher LF for children with CP (*M* = 10 N, SD = 4.7) compared to TD children (*M* = 8.8 N, SD = 1.1) and increased time to reach LF maximum value for children with CP (*M* = 1.9 ms, SD = 1.8) compared to TD children (*M* = 0.9 ms, SD = 1.5).

The **adaptation along trials** of the LF peak rate did not differ between groups or ages for either the dominant or non-dominant hand. A significant difference from zero was only found for the non-dominant hand in the younger TD group (adjusted *p*-value <0.001). This observation suggests a general tendency for constancy throughout the task, except for the younger TD children using their non-dominant hand, which modulates their LF peak rate.

The **amount of change between pairs of trials** using the relative change in the LF peak rate differed between groups for both the dominant hand (*H* = 7.08, adjusted *p*-value <0.01) and the non-dominant hand (*H* = 13.74, adjusted *p*-value <0.001). This observation suggests that the amount of change between pairs of trials varied according to the group, irrespective of the hand. A slope significantly different from zero was observed only for the non-dominant hand in older TD children (adjusted *p*-value = 0.002). This result indicates that the relative change in the LF peak rate differed explicitly for this group and condition.

**Figure 3:**
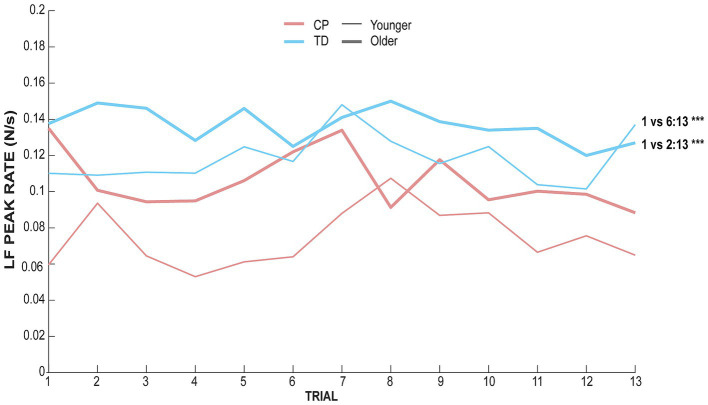
LF peak rate of each group and each age range along trials for the non-dominant hand. The red ink line represents data for children with CP whereas blue ink line represents TD children data. The thick line represents data for children aged between 11 and 17 whereas the thin line represents data for children aged between 8 and 11.

The **effect of trial** for every condition showed significant differences between trials only for the non-dominant hand in the TD group for both the younger (*χ*^2^ = 46.31, adjusted *p*-value <0.001) and the older group (*χ*^2^ = 36.03, adjusted *p*-value = 0.001). Post-hoc pairwise comparisons between each pair of trials are depicted in Figure 3, indicating that both younger and older TD children using their non-dominant hand showed adaptation to the task after a few trials. The younger TD children could adapt from the 6th trial since no effect of trials is observed after the 6th trial, whereas the older TD children adapt from the 2nd trial since no effect of trials is observed after the 2nd trial.

#### 3.1.2 Objective 2: association between feedforward control and white matter tracts

**Figure 4:**
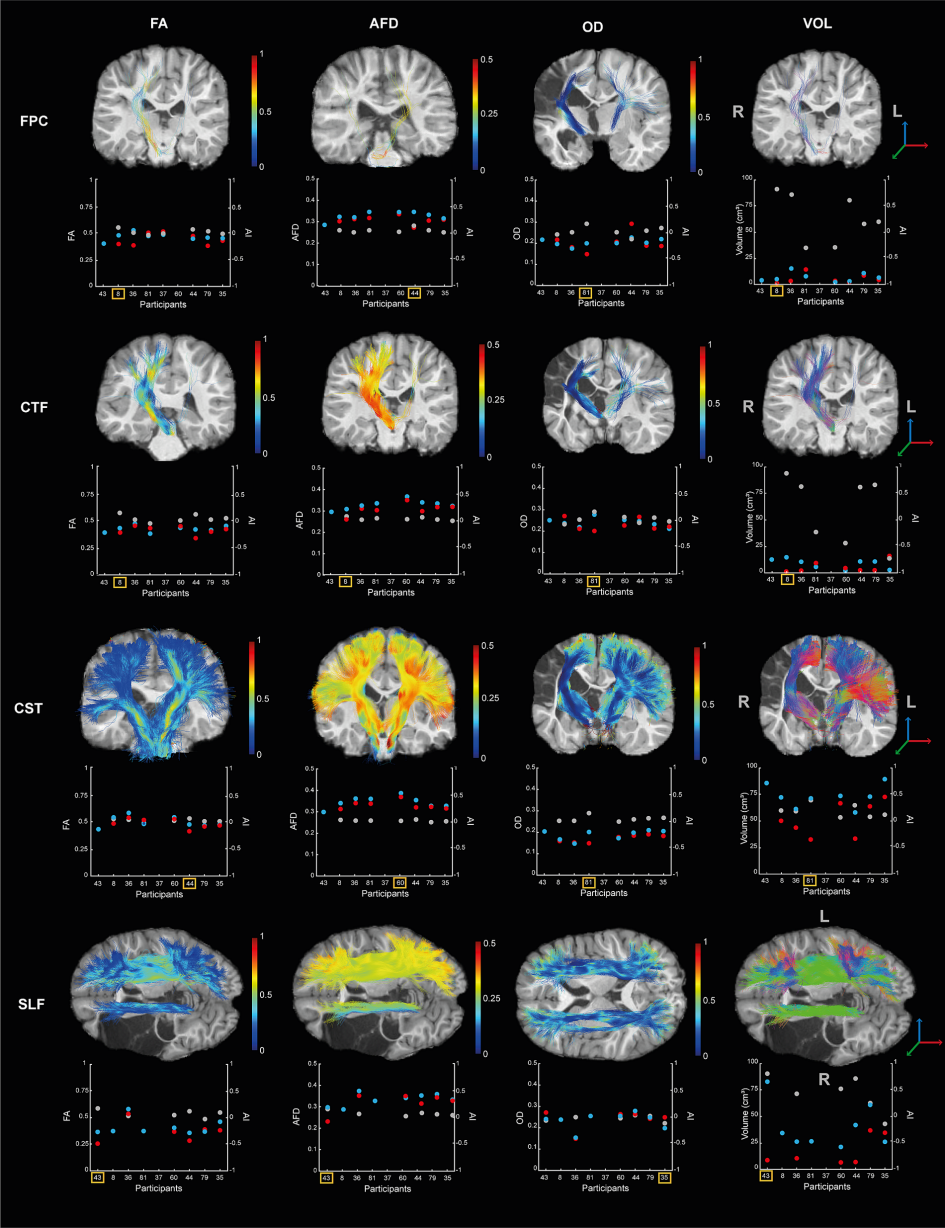
Diffusion metrics (i.e., FA, AFD, OD, and volume) for the four tracts (i.e., FPC, CTF, CST, SLF) by participants. Each scatterplot represents the data for the lesioned hemisphere (i.e., red dot) and the controlesional hemisphere (i.e., blue dot). The asymmetry indices are indicated in gray (i., right axis). The tracts of the participant showing higher asymmetry between hemisphere is displayed on their T1 image and indicated by the yellow square on the scatterplot. The colors of the tracts represent the metric variation along the tract.

**Figure 5:**
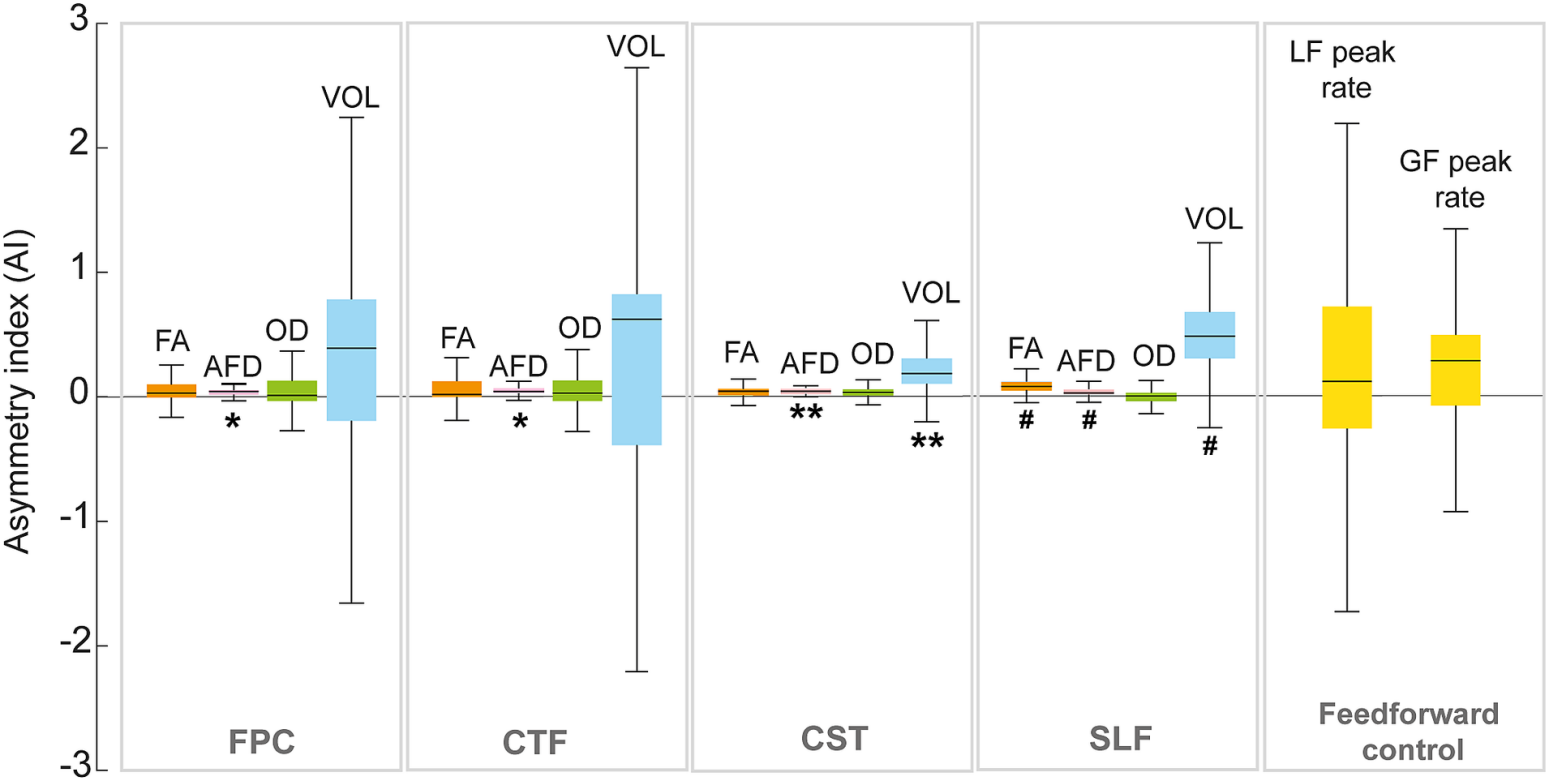
Median and interquartile range of the asymmetry index (AI) of the fronto-ponto-cerebellar (FPC), cerebello-thalamo-frontal (CTF), corticospinal (CST) tracts and superior longitudinal fasciculus (SLF) for the dMRI metrics (FA; fractional anisotropy, AFD; apparent fiber density, OD; orientation dispersion index). The y-axis represents the asymmetry index (AI). Significant differences from zero are indicated with ** (*p* < 0.01) and trend (i.e., *p*-value between 0.05 and 0.1) are shown with #.

It was possible to reconstruct all tracts on both sides in all participants, except for the FPC and CFT tracts on the lesioned hemisphere for one participant (S43) and of SLF in the lesioned hemisphere for two participants (S8 and S81). The tracts and the metrics are illustrated in Figure 4. Asymmetry of the FPC and CTF metrics and GF and LF peak rates asymmetry indices are displayed in Figure 5.

**Figure 6:**
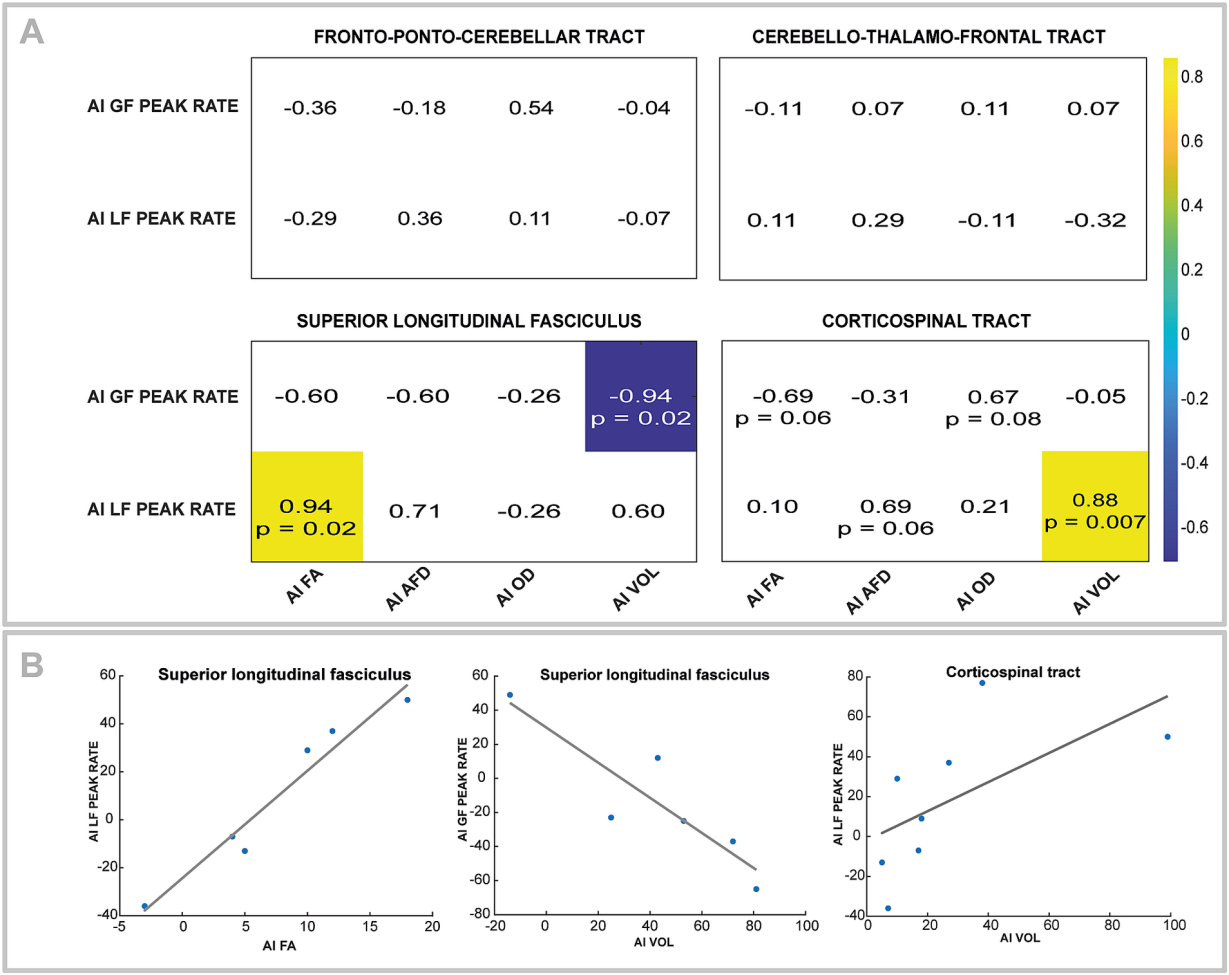
**(A)** Correlation matrix for asymmetry index (AI) of GF peak rate and LF peak rate with fractional anisotropy (FA), apparent fiber density (AFD), orientation dispersion index (ODI), and volume (VOL) for fronto-ponto-cerebellar and cerebello-thalamo-frontal tracts. All correlation coefficients are indicated, but only significant ones are colored. Trends (i.e., *p*-value between 0.05 and 0.1) are also noted. **(B)** Scatterplots and regressions line corresponding to the significant correlation in the A section.

No correlations were found between feedforward control and FPC or CTF tracts. Significant strong negative correlations were found between GF peak rate asymmetry and SLF volume asymmetry (*r* = −0.94, *p* = 0.02). For the asymmetry of the LF peak rate, a positive strong correlation was observed with asymmetry of FA of the SLF (*r* = 0.94, *p* = 0.02) and the CST volume asymmetry (*r* = 0.88, *p* = 0.007). Figure 6 shows the correlation matrix with *p*-value.

## 4 Discussion

The present findings demonstrated that children with CP exhibited the expected lateralized differences of force control compared to TD children, as indicated by lower GF and LF peak rates, this deficit being more pronounced in the younger group for the GF peak rate. The adaptation results were mixed. While no significant adaptation in GF peak rate was observed in either group, the LF peak rate adaptation was present only in TD children, specifically when using their non-dominant hand. Younger TD children adapted after the 6th trial, while older children adapted earlier, starting from the 2nd trial. Although feedforward control variables were associated with CST and SLF microstructural measures, no correlations were found with the corticocerebellar tracts, contrary to our expectations. This lack of association could suggest that corticocerebellar pathways may play a less critical role in feedforward control in children with CP.

The differences in the forces (i.e., GF and LF) peak rates for the non-dominant hand in children with CP can be attributed to either applying lower force or taking longer to apply the force. This effect is more pronounced in younger children with CP, likely due to motor developmental delays (74), while older children with CP show less pronounced feedforward control deficits that improve with maturation over late childhood (40, 41). Children with CP often take longer to apply forces, as reflected by an extended longer time to reach maximum raw force, likely compensating for sensory deficits by stimulating more mechanoreceptors (75–77). These differences may reflect feedforward control deficits as peak force occurs before sensory feedback is available (78, 79). However, it has been suggested that reduced forces peak rate in children with CP are indicators of their reliance on sensory input to adjust their grip and then initiate lifting (79, 80), controlling their movement through feedback control (81–83). Peripherical factors may also contribute since reduced forces peak rates are only present in the hemiplegic hand. The GF is associated with sensory and motor impairments in stroke patients (84), and the GF peak rate is linked to pinch strength and tactile discrimination in CP (15). The LF is influenced by muscle and joint function (25), and children with CP generally show reduced muscle activation in the hemiplegic arm (85). These findings likely indicate broader sensorimotor impairments and a combination of different factors rather than relying purely on feedforward control deficits (86).

This observation is further supported by the lack of correlations with corticocerebellar tracts (i.e., FPC and CTF), suggesting that the deficits in children with CP are more related to motor command and sensory integration in their deficits (i.e., CST and SLF). The correlations found for the SLF, involved in selecting grasp parameters based on object properties (58), underscores the role of sensorimotor tracts in refining the internal model, highlighting the importance of sensory feedback in updating the internal model (19). Adaptation across trials was only observed for the non-dominant hand of TD children confirming that they adjust their LF by the second lift (87). The absence of adaptation in children with CP may explain the lack of associations with corticocerebellar tracts. Adaptation involves forming internal representations based on sensory feedback from each lift, which guides future actions under similar conditions (16, 17, 79). In TD children, adaptation may be less pronounced in the dominant hand because internal models of object properties can transfer between hands (88). In the current study, TD children always first grasped and lifted the object using their non-dominant hand. Thus, forming the internal model is allowed by the first hand used in the task, but it requires proper integration of sensory information. Even children with CP can apply appropriate force with their non-dominant hand after initially grasping with their dominant hand, demonstrating that internal models can be shared between hands and is possible in CP when the dominant hand is used first, which allows sufficient sensory input for updates (89). Thus, the lack of adaptation in children with CP may be due to altered sensory integration related to object characteristics when starting with their non-dominant hand. This could lead to insufficient sensory input and/or altered processing, hindering the proper formation of internal models. These findings underscore the need for further investigation into sensory function in grasp and lift tasks.

The lack of adaptation in the GF peak rate for both groups was unexpected. GF is typically more closely related to object texture (90), while LF is influenced by object weight (91). The adaptation mechanisms and the potential for transferring internal models between hands, particularly in relation to texture, have not yet been studied (19). The GF peak rate showed less retention over days compared to the LF peak rate and is less critical for task success (88). Additionally, GF peak rate adaptation has only been observed in participants with normal wrist angulation; those with hyperflexion did not show adaptation (92). In our task, participants could either use a precision grip with the wrist aligned at finger level or a downward pinch with a flexed wrist. These different grip strategies have been shown to influence the feedforward control of GF (93). Standardizing wrist position might have allowed us to observe adaptation or at least account for its effects.

A surprising result concerns the strong negative correlation between SLF volume and the GF peak rate, indicating that lower volume asymmetry (i.e., more similar volume between hemispheres) is associated to greater asymmetry of the GF peak rate between hands. We expected the opposite correlation, as volume loss on the ipsilesional side is typically associated with more deficits (94). However, higher white matter tract volume does not always indicate better motor function. For instance, reduced white matter volume in children with autism is correlated with poorer motor function compared to TD children (95). This observation is especially pronounced in later myelinated white matter regions (96), and it has been shown that SLF volume matures later than most white matter tracts (97). In children with autism, the non-functional increase in white matter arises from global brain abnormalities that impair functionality, driven by microscopic abnormalities in cortical column organization that favor shorter connecting fibers over longer ones (95, 98). Similarly, children with CP have reduced long-range connections (94), suggesting that the increased volume may result from a reduced long-distance connection, such as those from the parietal and premotor cortices, but instead have higher local connections.

This study has a few limitations, the most notable being the small sample size of the CP group. Future research with larger sample sizes is needed to validate these findings, minimize errors, and increase statistical power (99). These preliminary results should therefore be interpreted with caution and serve as a foundation for future studies. Additionally, the limited clinical characterization in our study constrains the scope of our conclusions. Hence, the results may not be applicable to children with other types of CP or with more severe impairments. Given the potential relationship between sensorimotor deficits and feedforward control, a more detailed assessment of sensory deficits in children with CP is necessary (19). Testing these hypotheses in children with CP across a broader range of clinical and neurological profiles would strengthen the reliability of our conclusions. Regarding the MRI data, averaging the metrics along tracts may miss some variation. Future studies should consider segmenting tracts at multiple points (100). Finally, a last limitation of this study is the absence of imaging data for the TD group, which prevents us from determining whether the observed correlations are unique to the CP group or reflect broader developmental patterns. Future studies should include imaging data from typically developing children to better contextualize these findings.

## 5 Conclusion

In conclusion, this study reveals significant force control deficits in the non-dominant hand of children with CP, which may be attributed to impairments in feedforward control. The associations observed with the CST and the SLF, both essential for grip and lift force modulation, and the absence of correlations with corticocerebellar tracts suggest that broader sensorimotor processes, beyond pure feedforward control, may contribute to these deficits. Nonetheless, there is a need for future studies with larger and more diverse cohorts to understand better feedforward mechanisms both at the behavioral and neurological levels in children with CP.

## Data Availability

The raw data supporting the conclusions of this article will be made available by the authors, without undue reservation.
